# Psychosis Recovery Orientation in Malawi by Improving Services and Engagement (PROMISE) protocol

**DOI:** 10.1371/journal.pone.0293370

**Published:** 2023-11-30

**Authors:** Stephen Lawrie, Charlotte Hanlon, Lucinda Manda-Taylor, Martin Knapp, Martyn Pickersgill, Robert C. Stewart, Jen Ahrens, Judith Allardyce, Action Amos, Annette Bauer, Erica Breuer, Dennis Chasweka, Kate Chidzalo, Saulos Gondwe, Sumeet Jain, Demoubly Kokota, Kazione Kulisewa, Olive Liwimbi, Angus MacBeth, Thandiwe Mkandawire, Anthony Sefasi, Wakumanya Sibande, Michael Udedi, Eric Umar

**Affiliations:** 1 Division of Psychiatry, University of Edinburgh, Edinburgh, United Kingdom; 2 Centre for Global Mental Health, Health Service and Population Research Department, Institute of Psychiatry Psychology & Neuroscience, King’s College, London, United Kingdom; 3 Department of Psychiatry, School of Medicine, College of Health Sciences, Addis Ababa University, Addis Ababa, Ethiopia; 4 Department of Health Systems and Policy, Kamuzu University of Health Sciences, Blantyre, Malawi; 5 Department of Health Policy, London School of Economics and Political Science, London, United Kingdom; 6 Centre for Biomedicine, Self and Society, Usher Institute, College of Medicine and Veterinary Medicine, University of Edinburgh, Edinburgh, United Kingdom; 7 Tower Hamlets Early Intervention Service, East London NHS Foundation Trust, London, United Kingdom; 8 Pan African Network for Persons with Psychosocial Disabilities (PANPPD), Kamuzu University of Health Sciences, Blantyre, Malawi; 9 College of Health Medicine and Wellbeing, University of Newcastle, Callaghan, NSW, Australia; 10 Saint John of God (SJOG) Hospital Services, Lilongwe, Malawi; 11 School of Social & Political Science, University of Edinburgh, Edinburgh, United Kingdom; 12 Department of Psychiatry & Mental Health, Kamuzu University of Health Sciences, Blantyre, Malawi; 13 Zomba Mental Hospital, Ministry of Health, Zomba, Malawi; 14 Mental Health Users and Carers Association (MeHUCA), Kamuzu University of Health Sciences, Blantyre, Malawi; 15 Curative and Medical Rehabilitation Services Directorate, Ministry of Health, Lilongwe, Malawi; 16 African Mental Health Research Initiative (AMARI), Kamuzu University of Health Sciences, Blantyre, Malawi; Public Library of Science, UNITED STATES

## Abstract

Malawi has a population of around 20 million people and is one of the world’s most economically deprived nations. Severe mental illness (largely comprising psychoses and severe mood disorders) is managed by a very small number of staff in four tertiary facilities, aided by clinical officers and nurses in general hospitals and clinics. Given these constraints, psychosis is largely undetected and untreated, with a median duration of untreated psychosis (DUP) of around six years. Our aim is to work with people with lived experience (PWLE), caregivers, local communities and health leaders to develop acceptable and sustainable psychosis detection and management systems to increase psychosis awareness, reduce DUP, and to improve the health and lives of people with psychosis in Malawi. We will use the UK Medical Research Council guidance for developing and evaluating complex interventions, including qualitative work to explore diverse perspectives around psychosis detection, management, and outcomes, augmented by co-design with PWLE, and underpinned by a Theory of Change. Planned deliverables include a readily usable management blueprint encompassing education and community supports, with an integrated care pathway that includes Primary Health Centre clinics and District Mental Health Teams. PWLE and caregivers will be closely involved throughout to ensure that the interventions are shaped by the communities concerned. The effect of the interventions will be assessed with a quasi-experimental sequential implementation in three regions, in terms of DUP reduction, symptom remission, functional recovery and PWLE / caregiver impact, with quality of life as the primary outcome. As the study team is focused on long-term impact, we recognise the importance of having embedded, robust evaluation of the programme as a whole. We will therefore evaluate implementation processes and outcomes, and cost-effectiveness, to demonstrate the value of this approach to the Ministry of Health, and to encourage longer-term adoption across Malawi.

## Introduction

Psychoses–principally schizophrenia and related conditions, including affective psychosis–can be experienced as deeply distressing and disabling conditions. Around 50% of people with psychosis in High Income Countries (HICs) will have ongoing symptoms and social and occupational functioning difficulties (APA, 2013). In one particularly comprehensive study, conducted in Finland, the combined lifetime risk of all psychoses was around 3%, with a prevalence of 1% [1]. The global point prevalence of schizophrenia is estimated to be 0.3–0.6% [2, 3].

Duration of untreated psychosis (DUP) is a key issue of clinical and public health concern. A longer DUP of even a few weeks or months has been consistently linked to worse outcomes in terms of more severe symptoms, worse social, vocational, and overall functioning, and reduced quality of life. This has been reported in many countries around the world, including low- and middle-income countries (LMICs) within Africa [4, 5]. The most cost-effective interventions for schizophrenia in Nigeria include community-based treatment with older antipsychotic drugs plus psychosocial support or case management [6]. Clinical trials indicate that antipsychotic drug treatment leads to quicker symptom relief, reduces the risk of relapse, and probably improves quality of life [7], and that psychosocial interventions such as illness education and support reduce relapse risk [8]. Notably, however, antipsychotic drugs can have unpleasant adverse effects which reduce their acceptability [9].

Malawi is a sub-Saharan African country with approximately 15 million people under the age of 30. No studies of the prevalence of psychosis generally or schizophrenia in Malawi have been conducted, but the increased risk of schizophrenia among the young and those living in poverty [10] are reasons to think it may be higher than in HICs. The prevalence of HIV/AIDS, malaria, and cannabis use [11, 12] could mean that organic and substance-induced psychoses are particularly common. Epidemiological surveys also suggest that brief psychoses could be more common in LMICs such as Nigeria than HICs [13].

Mental health services in Malawi are centralised in a Ministry of Health in-patient unit in Zomba (Southern region) and an outpatient clinic in Blantyre (Southern region), and St John of God Hospitaller Service facilities in Lilongwe (Central region) and Mzuzu (Northern region). Specialist mental health staff are scarce. Across the whole country there are four psychiatrists, three clinical psychologists, and seven social workers who are based in these four tertiary facilities [12]. Secondary care services in district hospitals are staffed by limited numbers of Mental Health Clinical Officers, who are clinical officers with additional degree level mental health training, and psychiatric nurses. Primary care is provided by general nurses, general clinical officers and medical assistants. District Mental Health Teams operating in district hospitals and outreach clinics are able to diagnose and treat psychosis, and screen for medical causes such as HIV/AIDS, malaria and cannabis use [14]. However, primary health care facilities in communities are staffed by non-specialist staff with limited capacity to diagnose and treat.

Most people in Malawi experiencing distress or unusual experiences/behaviours that would correspond to ICD/DSM diagnoses consult traditional or religious healers, and receive herbal or spiritual remedies. Combined with low accessibility of alternatives, people with possible ‘psychosis’ are frequently brought late to healthcare service attention. Consequently, it is only people with the most severe forms of psychosis that come to the attention of mental health professionals, such that the median DUP is estimated to be 71 months [15]. The vast majority of people with psychosis in Malawi do not receive either pharmacological or psychosocial interventions, with a likely adverse impact on outcome, caregiver burden and increased risk of adverse experiences including homelessness, accidents and assaults [4, 16].

These limits to the capacity of existing health services in Malawi to provide care for people with psychosis and their families form the rationale for our research. It’s feasibility, in turn, relates to several notable strengths and initiatives within Malawi. These include the many researchers and institutions collaborating on this project, but crucially Health Surveillance Assistants (HSAs). These are secondary school graduates trained in health, located throughout Malawi at the village level, and primarily tasked with managing TB and HIV/AIDS. They typically serve a 1,000–2,000 population, and 10–20 HSAs are affiliated with each Primary Care Health Centre. In April 2020, the Ministry of Health launched a new policy to improve access to integrated quality mental health services, including training HSAs in mental health. We, therefore, plan to train HSAs to identify possible psychosis, use referral pathways, and provide community-based brief psychosocial interventions.

### Aims

The overarching goal of our research is to build on existing services and initiatives, to develop acceptable and sustainable psychosis detection systems and management pathways, and to improve outcomes for people with psychosis in Malawi. Our specific aims are:

1. To understand how perceptions and thoughts that would be recognised in diagnostic manuals as ‘psychosis’ are experienced and conceived of among people with lived experience (PWLE), caregivers, and HSAs in Malawi, and how these influence help-seeking and preferences for care;

2. To develop and validate a screening tool as part of a psychosis detection system in Malawi that is accurate, useful, and acceptable to PWLE, HSAs, and caregivers, and which facilitates engagement and reduces DUP;

3. To formalise a readily implementable psychosis management plan, emphasising community-level psychosocial interventions, with facility-based physical health care and receipt of antipsychotic medication, that is acceptable to PWLE, caregivers, HSAs and health workers;

4. To evaluate the implementation processes, effectiveness and cost-effectiveness of the psychosis detection and management system in improving outcomes prioritised by PWLE, caregivers, and health workers.

## Materials & methods

We will integrate the programme design and evaluation tool, Theory of Change (ToC), into the UK Medical Research Council (MRC) Guidance for Developing and Evaluating Complex Interventions [17, 18] to enhance multiple stakeholder engagement and embed our interventions in the local context [19], and to address implementation processes and outcomes throughout [20]. The PRIME project developing integrated district mental healthcare plans in Ethiopia, India, Nepal, South Africa and Uganda [21] used this approach and found it was helpful in (a) supporting systematic thinking around complex interventions, (b) making assumptions explicit early in the process, and (c) developing consensus around outcomes. Another advantage of this approach is the commitment of ToC to an ongoing process of reflection. This allows adaptation as barriers to implementation arise, and new evidence comes to light, requiring pathways to be changed and strengthened. This is also harmonious with the Quality Improvement activities that are valued in Malawi’s Government initiatives.

Our framework for PROMISE has four work packages (WPs): intervention development (WP1), feasibility testing (WP2), piloting (WP3), and implementation with evaluation (WP4) in an iterative process. Developing a context specific ToC within the first three WPs to underpin the psychosis detection and management system with stakeholders will ensure we have an explicit impact pathway, facilitate the translation of research into practice tools and allow a theory driven investigation of the mechanism(s) of action. Overall, this will ensure we have an intervention fit for context as developing contextually relevant interventions is a key emerging concern within global mental health.

A key aspect of our process is to work closely with PWLE and other stakeholders, collaboratively identifying what positive outcomes and what real world impact they want to achieve, to determine pathways through which change can be effected using available resources. WP1 will thus develop locally embedded plans and success indicators that are acceptable and feasible within resource constraints. This will allow us to incorporate evidence from other settings if relevant and helpful, and adapt existing evidence-based interventions as appropriate.

Our approach will support a flexible but robust approach to find effective ways of identifying and managing psychosis, without imposing methods that may not be relevant, practicable, or desirable to PWLE, caregivers, and health professionals in Malawi. This shared vision as to what is desired and how coordinated actions will bring that about will foster the motivation across stakeholders to sustain the intervention following the research.

### WP1—Development of a psychosis detection and management system

In WP1, we seek to better understand: how ‘psychosis’ is conceived and experienced; what idioms of distress and traditions of knowledge are used to articulate experience; how conceptions, experiences and environments influence help-seeking and management, priorities, and preferences for care; and, what outcomes are desirable among PWLE and their families. We also seek to enhance our understanding of these issues in relation to traditional and religious healers, as well as health-workers including HSAs, Primary Care Health Centre and District Mental Health Team staff. We will build on our existing collaborations and work conducted in Healthy Lives, Generation Malawi [22, 23] to do so.

#### Activities

The activities for WP1 will include exploring and seeking to understand how PWLE can be supported to contribute to our research: both as qualitative participants in WP1 itself and–through ongoing engagement with the research team–as stakeholders in the intervention and shapers of this. Methods associated with Participatory Action Research, such as Photovoice, will be used to go beyond a sole focus on discourse into the realm of perception and experience through photography. Photovoice will: enable people to record and reflect on their perceptions, experiences, and concerns; promote knowledge and facilitate open communication about psychosis and its impact on individuals and families through group discussion of images; and, reach and inform health policy makers to help bring about change [24]. WP1 will produce research findings that will result in the development of a prototype detection and management intervention within 18 months.

To enhance what is already known about experience of psychosis within Malawi and elsewhere in Africa, a narrative, scoping review will be carried out on qualitative studies of madness/psychosis in Africa to date. Thereafter, WP1 activities will occur in two districts: one in Central (Salima) and one in Southern (Chiradzulu) Malawi. Within each district, key informants will be drawn from both an urban/peri-urban area and a rural setting.

The key informants are PWLE and their families, other formal and informal caregivers, traditional and religious healers, and healthcare staff (in particular, HSAs)—see Table 1. A total of 12 PWLE and 12 carers will be purposively sampled, with the assistance of Peer Support Workers (PSWs) from the Mental Health Users and Carers Association (MeHUCA) of Malawi and the Chiradzulu District Hospital (CDH) mental health clinicians. The latter will identify those with psychosis in community settings, including some with experience of secondary and tertiary care and prison, who will be invited to participate.

**Table 1:** WP1 activities to co-produce a psychosis management and detection system.

**Research questions**	**Methods**	**Key informants/participants**	
		**Community**	**Healthcare**
*What does the scientific literature say about the perception of psychosis in LMICs*?	Systematic reviews	**-**	
*What is the lived experience of people living with psychosis*?	In-depth interviews and focus groups (n = 24); Participatory empowerment using Photovoice (n = 12)	PWLE Family/caregivers	**-**
*What are the needs*, *perceptions of mental health and views of involvement in identification and referral among HSAs*?	Survey methods (n = 40)	-	HSAs
	In-depth interviews and focus groups (n = 20)	-	HSAs
*What are stakeholders’ views on psychosis detection and management systems*?	Key Informant Interview and Focus groups (n = 28)	PWLE Family/caregivers Traditional Healers Religious Healers	HSAs PHC staff DMHT staff
*What is a contextually appropriate and feasible programme theory for a psychosis detection and management system in the two districts in Malawi*?	Theory of Change workshops 1&2 (n = 20 per district)	PWLE Family/caregivers Traditional Healers Religious Healers	HSAs PCHC staff DMHT staff Government representatives

Key: PCHC = Primary Care Health Centre; DMHT = District Mental Health Team

Photovoice data collection will include four stages. The first will involve consent and training. The second stage consists of individual meetings where participants verbally reflect on each photograph using the SHOWED technique, previously used in Photovoice research [25]. The third stage is a group discussion where participants present selected pictures using the SHOWED technique. The other participants engage in dialogue to share similar or differing stories of experiences during the photograph presentations and ask each other questions. The final stage is a stakeholder meeting where health providers will be invited to hear the presentations. The stakeholders will be encouraged to ask follow-up questions to shed light on implementation strategies.

We will also conduct qualitative and quantitative research with the available sample of 40 HSAs, including self-completed questionnaires: the Community Attitudes toward Mental Illness scale, the WHO mhGAP pre- and post-knowledge test, and a confidence in identifying mental disorders questionnaire which have been used before in Malawi [14, 26]. Both data types will support a longitudinal understanding of HSA perceptions of mental health, experiences, and needs as health-workers, and perceived obstacles and enablers to supporting PWLE of psychosis and their caregivers. In each district, a social science team will conduct focus groups and in-depth interviews with a smaller sub-sample of 10 HSAs. Respondents will be purposively sampled, taking account of seniority, gender, and age. These focus groups, interviews, and surveys will be repeated each year of the project to understand ongoing experiences of HSAs, changes in their perspectives, and views on the intervention.

Additional qualitative data will be gathered from up to 28 key informants from the community and the health care system to understand diverse views on psychosis detection and management systems. This will be completed through key informant in-depth interviews and focus groups with: PWLE; family, and other caregivers; traditional and religious healers; and, HSAs and healthcare staff.

All the qualitative data will be audio-recorded, transcribed, and translated from Chichewa (the main language used in Malawi) to English. We will employ a thematic content analysis. The data analysis will involve several stages, including submersions, coding, and categorising to develop themes, helping inform the ToC development.

The findings from the mixed-methods approach will raise awareness and motivate change through the development of a psychosis detection and management system. Co-production with stakeholders will include collaboratively identifying what real-world impact they want, charting causal pathways through which change can be achieved in their context using available resources, and potential areas for psychosocial intervention consistent with cultural norms. The resulting ToC Roadmap will identify actionable findings relative to implementation strategies and stakeholder data to understand feasibility. This will inform the draft of a psychosis detection and management manual in Chichewa, enhanced by the findings of the literature reviews and recovery narratives from PWLE. We anticipate that those recovery narratives will also be able to be used, subject to informed consent, as a form of social contact with PWLE to reduce stigma.

### WP2—Feasibility testing of the psychosis detection and management system

A diagnosis of psychosis can help explain unusual experiences and behaviour to those affected and others, increase tolerance, reduce distress and stressful social obligations, and have functional value towards recovery [27]. This may be especially so if moral or supernatural causal attributions are prevalent, as in Malawi. However, a diagnosis may also be perceived as a limiting label, devalue pre-existing understandings of the experience, and increase stigmatisation for the individual and the family. More generally, psychiatric diagnosis can act as a vehicle for conceptions of self and society that emphasise cultural norms within HICs, and require careful consideration when used in the majority world [28, 29]—not least given concerns about biomedicalisation in global mental health [30]. Work conducted in some LMICs about the impact of a diagnosis of psychosis suggests that paced, individualised, and collaborative approaches are most valued [31]. These issues will be discussed with HSAs as part of their training. Our approach will be to encourage HSAs to take time and seek additional information if required to detect possible/probable cases.

*Activities*. WP1 will generate a ToC Roadmap and a draft of a psychosis detection and management manual. WP2 activities, therefore, focus on: a) translating these into a finalised manual; b) development of feasibility measurement methods; c) understanding HSA views of these; d) supporting HSAs to use the materials and procedures with a group of adults; and e) reviewing the final prototype manual and procedures.

WP 2 feasibility testing will be done in the sites where WP 1 was completed. This will allow feasibility testing in locations where community support has already been established. Given that the draft manual will have implications for both the community and the healthcare system, the key informants in this WP are PWLE and their caregivers, traditional and religious healers, HSAs, and other healthcare staff (see Table 2 below).

**Table 2:** Feasibility testing of the psychosis detection and management system in WP2.

**Research questions**	**Methods**	**Key informants/participants**	
		**Community**	**Healthcare**
*How can the feasibility of the psychosis detection and management system be measured in context*?	ToC workshop 4 (n = 10 [EB1] per district)	-	HSAs PHC staff DMHT staff Government representatives
*What is the feasibility of the psychosis management and detection system in Salima and Chiradzulu*?	ToC workshops 3 and 5 (n = 20 per district)	PWLE Family/caregivers Traditional Healers Religious Healers	HSAs PHC staff DMHT staff Government representatives
	Survey methods (n = 40)		HSAs
	Feasibility testing	People with possible psychosis (n = 20)	HSAs (n = 10)

Key: PCHC = Primary Care Health Centre; DMHT = District Mental Health Team

The first WP2 ToC workshop (ToC 3 overall) will review our understanding of the ToC Roadmap, review the draft manual, and consider the implementation strategies that will be needed to successfully deploy the intervention.

The second WP2 ToC workshop (ToC 4 overall) will include healthcare colleagues only, considering the healthcare elements of the intervention, to review the implications of the draft manual for the delivery of current services, and to develop feasibility and fidelity-to-manual measures. The third and final ToC of WP2 workshop (ToC 5 overall) will consider the final prototype manual and procedures alongside the feasibility testing data.

Given the HSAs are the group of professionals we are focussed on in this programme, their views of the draft manual and procedures will be critical. We will qualitatively and quantitatively re-survey 40 HSA’s (from those surveyed in WP 1). This is also two-thirds of the cohort of HSA’s who will be involved in the roll-out of the approach in WP3. This continuity reinforces the innovation pathway, by strengthening engagement with this group of professionals through early sight of draft materials and facilitating co-production via shared perspectives—thus shaping the final prototype manual and materials.

We will train 10 HSA’s in one of the two districts in WP1, in both the urban and rural setting, to use the draft manual and procedures to set up the first deployment of the approach. This will also require the training of Primary Care Health Centre and District Mental Health Team staff in the psychosis detection and management system, and in particular to support and supervise HSAs. This approach will then be ‘beta-tested’ within the regular working day of HSAs for two months to test it in practice. Any challenges will be logged in a risk register and solutions found with the Community Advisory Board. We plan to conduct feasibility testing on 20 people with possible psychosis, referred to them through the community or from healthcare staff.

### WP3—Piloting of the psychosis detection and management system

There is clinical trial evidence to support the efficacy of illness education and support, family interventions and antipsychotic medication, in acute and established psychosis, in Ethiopia, India and other LMICs [32–34]. There is also evidence that informal healthcare providers in some LMICs can be supported to collaborate with facility-based healthcare workers [35], and that this can be cost-effective [36]. In Malawi, we will build upon three recent initiatives. Wright & Chiwandira [37] have shown that it is possible to build capacity for community mental health care, including psychosis, with a district-wide task-sharing intervention with HSAs. Kokota et al. [14] demonstrated that materials from the WHO mhGAP—a popular if not uncontroversial tool for guiding mental health care in low resource settings [38]—can increase primary healthcare workers’ knowledge and confidence in providing care for people with mental health conditions in Mulanje, southern Malawi. The Malawi Quick Guide to Mental Health (www.smmhep.org.uk) was published in 2020 to provide practical information for all healthcare workers in the assessment and management of mental disorders.

#### Activities

The key activities in WP3 will be to pilot the prototype manual and procedures of the psychosis detection and management system to N = 268 people to identify an anticipated N = 150 who screen positive for psychosis. This will be carried out sequentially, district by district, in the two districts involved in WPs 1 and 2 (Salima and Chiradzulu) and a third district (Ntcheu), which is equidistant between the others. The key informants in this WP are PWLE and their families, formal and informal caregivers, traditional and religious healers, HSAs, and clinic staff in Primary Health Centre and District Mental Health Teams. A process of engagement needs to be developed in the new Ntcheu district involved in WP3. This will be completed through ToC 6 workshop with PWLE and their families, and the other stakeholders above. Table 3 below gives an overview of WP3 activities.

**Table 3:** WP3 piloting of the psychosis detection and management system in 3 districts.

**Research questions**	**Methods**	**Key informants/participants**	
		**Community**	**Healthcare**
*What are the contextual adaptations required to implement the psychosis detection and management system in Ntcheu*?	ToC workshop 6 (n = 20 in Ntcheu)	People with lived experience Family/caregivers Traditional Healers Religious Healers	HSAs PHC staff DMHT staff Government Rep
*To what extent can HSAs trained in the psychosis and management tool accurately detect and refer people with psychosis to further care*?	Validation study (n = 268 screened)	People with possible psychosis	**-**
*Can the psychosis detection and management tool be delivered with fidelity by HSAs*?	Fidelity assessment (n = 60)	-	HSAs
*How did people with psychosis experience the detection and management process and what are the views of their diagnosis*	Qualitative interviews (n = 20 across three districts)	People with psychosis	-
*How did HSAs experience the detection and management process*? *How have their views on people with psychosis have changed*?	Qualitative interviews (n = 20 across Salima and Ntcheu)	-	HSAs
*What is the impact of the psychosis management and detection tool on service utilisation*?	Review of district health data	-	-

We will train 20 HSAs in three districts (n = 60) in the prototype psychosis detection and management system, to (a) screen for psychosis and engage with a management system, (b) provide community-based brief psychosocial interventions, and (c) use referral routes to consultations with a mental health specialist when the presentation is complex. This will situate the HSAs explicitly within a healthcare pathway and equip them with the knowledge and skills to support earlier detection and delivery of elements of care at the village level. We will train Research Assistants (RAs) to administer the Present State Examination gold-standard diagnostic interview as part of the WHO’s SCAN (WHO, 1997). This will be used to screen positive and negative cases (validation study). The RA will facilitate community-based psychosocial interventions for people who screen positive for psychosis. Inter-rate reliability of diagnosis and outcome measures at baseline will be established.

#### Validation study

A power calculation, using Python, identified that we will need to screen 268 people with ‘possible’ psychosis to identify 162 ‘true positives’ to have at least 80% power (alpha 0.05) to detect a change percent value of specificity of the diagnostic tool from 80% to 90%. Initial case-finding of people with ‘possible’ psychosis will result from awareness-raising in the community and from training HSAs. People with possible psychosis will then be assessed by HSAs using the screening tool. All people who are considered to have psychosis based on the screening assessment (‘probable’ psychosis) and a random sample of screen negatives will undergo the gold standard diagnostic assessment administered by the RAs. The RAs will be masked to the outcome of the screening test. The gold standard diagnosis will include any diagnosis within the ‘schizophrenia or other primary psychotic disorders’ section of ICD-11, and affective psychosis, but will exclude organic or substance-induced psychoses. We will investigate sensitivity, specificity, positive and negative predictive values. The key concerns are to minimise false positives of a definitive psychosis diagnosis and to detect those with ongoing (not brief) psychosis that would benefit from an intervention that can be provided. We will use multivariate analyses to investigate possible predictors of inaccurate diagnosis (e.g. gender, educational level, age) to inform further implementation.

#### Evaluating impact on service utilisation

We will conduct an interrupted time series study to evaluate the impact of the detection component (case-finding, diagnosis, referral, and engagement) of the psychosis detection and management system on service utilisation. Following the HSA screening assessment, people with probable psychosis will be referred to the appropriate facility in the care pathway, depending on severity. We will establish a simple, paper-based, case registration system in all the potential referral facilities, including private sector facilities, at least three months prior to initiating case-finding in that district. This will create sufficient baseline data time points in the pre-intervention period to allow us to assess levels of service utilisation for people with psychosis (new and existing) prior to the intervention and during the implementation of the detection system. This method has been used previously in Malawi to investigate the impact of an intervention on general mental health contacts with facilities [14]. We will use segmented linear regression using aggregated monthly utilisation data to assess whether the detection system leads to a significant increase in utilisation of each type of service beyond pre-intervention trends [39].

#### Deployment of the prototype manual and procedures

The HSAs will deliver the agreed protocol. We will conduct in-depth interviews with 20 people diagnosed with psychosis to understand their experience of the process and their perspectives on the diagnosis. We will also repeat the baseline survey (done in WP1 and 2) alongside in-depth interviews and focus groups with the HSAs who have been through the training and deployment to understand their views of the process and psychosis.

#### Fidelity and feasibility

The fidelity and feasibility approaches and measures will be developed from ToC 1 & 2 roadmaps and formally considered in a structured way in ToC workshop 4 and 6. However, it is anticipated that the indicators identified in Table 4 below will form part of the feasibility testing structure. Both qualitative and quantitative measures will be taken to establish fidelity to the programme procedures. HSAs will be purposively selected across a range of levels of engagement with the detection intervention, gender, and years of experience.

**Table 4:** WP3 anticipated feasibility testing structure.

**Programme Procedures**	**Quantitative**	**Qualitative**
Numbers screened, eligible, consented, completed screening/diagnostic assessment	Research log	
Number who lack capacity	Research log	
Refusal rate	Research log	
Acceptability of procedure		In-depth interviews with subsample of 20 HSAs
The number who were diagnosed with psychosis	Research log	
Experience of process		In-depth interviews with a subsample of HSAs and 10 PWLE
The burden of assessment procedures	Duration of assessment	
Health Surveillance Assistants: changes in their views about psychosis detection and management	Repeat baseline survey	
Health Surveillance Assistants’ views about processes and tools	Enhanced survey with screening/diagnosis questions	

### WP4—Evaluation of the psychosis detection and management system

Given the ethical issues associated with randomised controlled trials in this context, in particular that a control group would equate to no care for some or even most people [40], we have opted for a sequential implementation (WP1, 2 & 3) and a quasi-experimental statistical evaluation of outcome (WP4). Intervention cohort studies without a control group have been shown to be beneficial when creating a control group is unethical [41]. This also has the practical advantage of allowing us to support strengthening primary care and the management by District Mental Health Teams of people with psychosis as we identify them in each district. It also allows for iterative improvement of the intervention, replicating what happens in routine settings. To address ’maturation’ effects (e.g., change in staff performance due to the evolution of clinical skills) and Hawthorne effects, we will employ a 12-month intervention and observation period. We will keep a log of any time-related changes in service delivery and integration into primary care during the process. This will allow us to closely monitor any secular trends, such as changes in local healthcare provision or MoH policies, that might impact upon delivery of intervention or outcomes. In addition, we will continue qualitative and quantitative data collection with the HSAs surveyed throughout to give us insight into potential mechanisms.

#### Activities

*Intervention cohort study without controls / pre-post sequential group design*. The analysis of individual outcomes will use appropriate descriptive statistics to present the outcome in each district as well as the overall statistic for all three districts. The outcome data will be stratified by health equity indicators (sex, age category, urban/rural residence) and by drop outs and loss to follow up. A sample size of 150 will be sufficient to detect a pre-post improvement of 20% overall (across the four sub-scales) of the WHOQoL primary outcome measure with a power of 90% (alpha = 0.05). The data will have a three-level structure with repeated outcomes measures collected with individuals clustered within three separate assessment centres. It is reasonable to assume that different data from people with psychosis in the same centres will be correlated and measurements on the same person even more highly correlated. To handle this data structure we will use Mixed Models (including fixed effects and random latent effects) using the appropriate model for the distribution of outcome. For the continuous scaled primary outcome WHOQoL, a linear mixed effect model will model change before and after intervention to estimate mean difference as well as the moderating effects of key health inequity measures (gender, urban/rural residence, socio-economic status) and process indicators (e.g. engagement with facility-based and community-based interventions).

The final selection of outcome measures for the evaluation will be informed by PWLE of psychosis and other stakeholders, and Theory of Change roadmaps (ToC 1; ToC 2; ToC 3; ToC5) inclusive of agreed indicators of success. Quality of life is a valued outcome for both psychosocial interventions and antipsychotic medication, and is therefore the current proposed primary outcome measure. The range of potential measures are indicated in Table 5 below with outcome measures carried out at baseline (WP3) and at 6 and 12 months (WP 4). This allows for the incorporation of other measures prioritised by PWLE and other stakeholders, such as food insecurity [42], and will give enough time for participants to engage with the innovation to investigate feasibility, fidelity, cost-effectiveness and outcomes.

**Table 5:** WP4 anticipated outcome measures.

**Focus**	**Measure used to capture focus**	**Baseline (WP3)**	**6 months (WP4)**	**12 months (WP4)**
Primary outcome PWLE				
Quality of life	WHOQoL	x	x	x
Health Economics				
Disability	DALYS (symptom data)	x	x	x
Quality of life	WHOQoL	x	x	x
Secondary outcome				
Duration of untreated psychosis	DUP	x	x	x
Symptom Severity	Positive and Negative Syndrome Scale (PANSS)	x	x	
Family Burden	Family Burden Interview Schedule (FBIS)	x	x	
Health and disability	WHO Disability Assessment Schedule (WHODAS)	x	x	

#### Economic analysis

The economic analysis, which will take a societal perspective, will establish: (1) the cost of the intervention, which will include the cost of the HSA training in, and implementation of, case detection and management; (2) the impact of the intervention on service-related and wider societal costs (cost consequences); (3) the cost-effectiveness of the intervention. For the costing of the intervention, the full costs will be calculated in the three districts, covering capital elements, staff time, consumables, and all other running costs. For establishing cost consequences, we will include costs associated with health and any other services (informed by WP3), out-of-pocket payments by individuals and families, productivity losses by individuals and families from disrupted employment, and the estimated value of unpaid support. For establishing cost-effectiveness, we will compare the cost of the intervention and associated cost consequences against the outcomes. The outcomes to be used in the economic analysis will depend in part on activities in other WPs but will include change in Disability Adjusted Life Years (DALYs) based on the Global Burden of Disease calculator to allow comparison with other cost-effectiveness studies. Whilst the main analysis will be from a societal perspective, in a secondary analysis we will look at cost-effectiveness from a health system perspective.

For establishing costs and consequences, data will be obtained at each of the data-collection points by asking patients (retrospectively) about service contacts, employment status, support from family and caregivers, and relevant personal expenditures (e.g. payments for services). We will compare these patient-reported service use patterns with data on impacts on service utilisation obtained from services themselves in WP3 to establish best possible estimates. Unit costs will be assigned to service utilisation data. These unit costs attached to service utilisation data will be drawn from extant sources and relevant previous research in Malawi, but it is likely that we will need to calculate most unit costs anew (for example, based on budget and salary information). In addition, we will use observations and questionnaires to establish the time that HSAs spend on the training and delivery of the intervention, and gather administrative data if needed. Lost productivity will be valued using data on employment disruption, earnings and care-giving or other household/community activities, applying human capital and opportunity costing approaches as appropriate; carer inputs will be valued on opportunity cost principles (including lost earnings).

To analyse the cost-effectiveness of the intervention, we will first calculate how costs change between baseline and follow-up: to show the resource consequences of the intervention across different budgets/sectors (healthcare, employment, individual households), and so inform judgements about future affordability. Cost-effectiveness analyses in this pre-post sequential group design will bring together the estimated costs and, in turn, data on the primary outcome (WHOQoL) and DALYs calculated from disability weights from the GBD/GHDx tool (http://ghdx.healthdata.org/gbd-results-tool). Incremental cost-effectiveness ratios will be computed under the assumption that baseline scores continue unchanged to follow-up, and that costs remain unchanged too. We will plot cost-effectiveness acceptability curves generated from bootstrap analyses. Discounting will not be needed as the evaluation does not extend beyond one year. Comparisons can be made with cost per DALY averted thresholds from [43] and other sources [44].

We will additionally conduct cost-consequences analysis, setting out changes in costs (total and component) alongside changes in all outcome measures. This more descriptive approach can be helpful to policy-makers when considering complex interventions of the kind developed in this programme. Sensitivity analyses will explore the impact of varying assumptions about key cost calculations (service unit costs, carer time, lost productivity), disability weights, extrapolation of baseline outcome scores and costs as the counterfactual.

#### Data management

The identifiable paper records (i.e. consent forms) will be kept in a locked cabinet, in a locked room, at KUHeS in Blantyre for the duration of the research project (5 years). Only the research team will have access to these. Consent from the participants will be sought for this.

All interviews will be audio-recorded, transcribed, and translated. The data will be obtained using a standard digital recorder, kept on researchers’ persons at all times thereafter, and then securely uploaded to a password-protected KUHeS university server space. Once audio recordings have been transcribed, the original audio recordings will be destroyed.

Qualitative and quantitative data from the study will be de-identified by a unique study number. That data will need to be shared between where it is stored at KUHES and researchers at the University of Edinburgh. It will, however, only be sent in an encrypted form via a secure university file-sharing platform, and with participant consent.

Qualitative outputs will require direct quotations from participants. These will be de-identified, and publication will be explicitly consented. Photovoice uses visual aids, as photographs, to communicate people’s perspectives—here, of psychosis. Subject to informed consent, some of these (and people’s narratives of psychosis) could be published, anonymously, within academic outputs and for purposes of public engagement (e.g., on any project website).

De-identified data will be kept for a period of 5 years after the study has ended and will be securely archived (again, subject to consent) on the University of Edinburgh’s DataShare platform for future research studies. Future research may be done by the current research team or by other research teams working in other countries. Only participant de-identified records, which do not have identifiable information, will be stored and shared with other research teams.

### Ethical considerations

The study will be conducted in accordance with the principles of the International Conference on Harmonisation Tripartite Guideline for Good Clinical Practice in addition to the principles of the ethics committees who have reviewed and approved this study. Approval was granted by the Edinburgh Medical School Research Ethics Committee (reference 22-EMREC-044) in November 2022, and by the Malawi College of Medicine Research and Ethics Committee (COMREC reference P.03/23/4034) in May 2023. Recruitment and Safeguarding Standard Operating Procedures (SOPs) have been developed from those used in other psychosis studies in LMICs.

Consent will be gained from each participant by a member of the research team and recorded on a consent form. If the person is not literate, a thumbprint will be obtained instead of a signature. In addition, an independent witness who is literate will sign to confirm that the information was read out as per the information sheet. Potential participants will be fully informed about the assessments in both the Participant Information Sheet and through discussions with the research team. It will be made clear that participation, or not, will not in itself lead to treatment, or not. Some participants may be patients of the three consultant psychiatrists in the research team, but they will not be involved in the decision to consent or not. Some people with psychosis may lack capacity to consent but they will be included if they express a clear willingness to participate and a carer gives permission, as they are representative of those who could receive the intervention if it works and the Malawi MoH implement it. If they regain capacity, they will only continue in the study if they give informed consent.

There is a risk that some participants could become distressed discussing their personal experiences and the potentially upsetting topics of psychosis, childhood maltreatment, and intimate partner violence. These risks will be minimised by recruiting experienced staff to ask such questions and telling people they do not have to discuss them if they do not want to and/or can stop at any time.

There is also a risk that people who screen positive for ‘possible psychosis’ and interview as ‘probable psychosis’ regard themselves as definitely diagnosed before a formal diagnostic interview has been done by the RAs. We will minimise this risk by clear written communication with terminology and content agreed with our Lived Experience Advisory Panels (LEAPs). There is also a concern that some people in those groups could benefit from intervention that is not available to them. Our LEAPs will input into development and implementation of the interventions in each district, and knowledge of what support is available in the community. Tailored information for possible / probable / confirmed psychosis will be available at a minimum.

### Status

The PROMISE study has been funded by Wellcome for 5 years, from 2022–27.

## Discussion

### Strengths and limitations

PROMISE is an innovative implementation science project designed to improve psychosis services and outcomes, and if so to understand how that was achieved. Of particular note are the principles of how we will engage with PWLE through the process, including partnership in co-design, and building networks and capacity. The psychosis awareness, diagnostic, and therapeutic materials that we develop could contribute to facilitating a contextualized roll-out of similar models of care in other LMICs in (sub-Saharan) Africa and beyond.

From the perspectives of guideline bodies that prefer efficacy to be evidenced through randomised controlled trials, the fact that we do not use this approach will be judged as the primary limitation of the work proposed. As discussed in WP4 (above), however, this would arguably be unethical where those in the control arm received no treatment at all. Our quasi-experimental sequential implementation design will allow for iteratively strengthening services and improving the intervention in each district.

There could also be tensions between an agreed research plan between ourselves and our funder, and what PWLE and others in Malawi may actually want–not least in having a specified primary outcome, to allow a power calculation, which may not be what those in Malawi value most. We acknowledge this in our ‘anticipated’ feasibility testing structure and outcome measures (see Tables 4 and 5), which are at the very least open to additions. It will be important for us to monitor and work with any tensions that arise through: regular, open, and frank engagement between researchers and other stakeholders; adjusting our workplan where feasible and if appropriate in response to external engagement; working to ensure an alignment in expectations between different stakeholders; and acting as a conduit for the communication of any concerns by PWLE to our funder and to healthcare providers.

### Dissemination plans

We will build community capacity and sustainability by increasing the awareness of psychosis. We will develop knowledge exchange materials specifically for the HSA training initiative. Community leaders and PWLE will be stakeholders from the start and will shape impact activity to promote our approaches, including action-orientated practical steps to encourage other community leaders to integrate our findings into their communities. The materials we use will be made relevant to government, commissioners, managers and practitioners by customising messages. This will result in a range of outputs which will be ‘refreshed’ throughout and after the funding period.

All materials developed will be free at the point of access hosted on the PROMISE website, and advertised through collaborators’ networks. This psychosis detection and management approach will be developed to be sustained and expanded over the longer term following the project. The research team is in close collaboration with the Ministry of Health, and opportunities will be sought to harmonise activities to secure governmental support. If we provide evidence of cost-effectiveness they will have a strong case to advocate the Malawi government support roll-out.

### Risk-reducing steps

Arguably the greatest threat to the project would be a failure to engage with PWLE and HSAs but we have successfully recruited and secured input from PWLE before in Malawi, our LEAPs provide ongoing relationships with many PWLE, and Kokota has previously worked closely with HSAs [14]. WP1 focusses on stakeholder engagement with resources for different approaches that may suit different people and backgrounds. In acknowledgement of the expanded HSA role and research administration, the grant will fund the HSA’s time and expenses; but, in the interests of long-term sustainability we will not pay them to participate in PROMISE or for assessments. Some HSAs are likely to be naturally interested and enthusiastic, and could become champions for this study and the future.
